# *Ex situ* Synthesis and Characterization of High Strength Multipurpose Bacterial Cellulose-*Aloe vera* Hydrogels

**DOI:** 10.3389/fbioe.2021.601988

**Published:** 2021-02-03

**Authors:** Mazhar Ul-Islam, Furqan Ahmad, Atiya Fatima, Nasrullah Shah, Somayia Yasir, Md. Wasi Ahmad, Sehrish Manan, Muhammad Wajid Ullah

**Affiliations:** ^1^Department of Chemical Engineering, College of Engineering, Dhofar University, Salalah, Oman; ^2^Department of Mechanical and Mechatronics Engineering, College of Engineering, Dhofar University, Salalah, Oman; ^3^Department of Chemistry, Abdul Wali Khan University Mardan, Mardan, Pakistan; ^4^Department of Industrial and Manufacturing Systems Engineering, Kansas State University, Manhattan, KS, United States; ^5^Department of Biomedical Engineering, Huazhong University of Science and Technology, Wuhan, China

**Keywords:** composites, mechanical properties, adsorption, *Aloe vera*, bacterial cellulose, biocompatibility

## Abstract

The innate structural and functional properties of bacterial cellulose (BC) have been greatly improved by developing its composites with other materials for its applications in different fields. In the present study, BC-*Aloe vera* (BCA) gel composite with high tensile strength was *ex situ* developed and characterized for its potential applications in environmental and medical fields. FE-SEM micrographs showed the impregnation of *Aloe vera* gel into the fibril network of BC. The dry weight analysis showed the addition of 40 wt.% *Aloe vera* contents into the BC matrix. The addition of *Aloe vera* resulted in a 3-fold increase in the mechanical strength of BCA composite. The critical strain or stress concentration points were accurately identified in the composite using a three-dimensional digital image correlation (3D-DIC) system. The BCA composite retained water for an extended period of up to 70 h. The BCA composite effectively adsorbed Cu, Co, Fe, and Zn metals. Moreover, the BCA composite supported the adhesion and proliferation of MC3T3-E1 cells. The findings of this study suggest that the developed BCA composite could find multipurpose applications in different fields.

## Introduction

Bacterial cellulose is a natural polymer produced by a class of acetic acid bacteria (Khan et al., 2020; Skiba et al., 2020; Ul-Islam et al., 2020b) and cell-free systems (Ullah et al., 2015). Both the microbial and the cell-free systems utilize different sugar sources and produce β-1,4-glucan chains, which are protruded extracellularly through the pores (i.e., terminal complexes) present in the cellular membrane, and form high ordered structures (Kim et al., 2019). The exciting structural, physico-chemical, mechanical, thermal, and biological properties of BC enable it to find applications in biomedical (Curvello et al., 2019; Ul-Islam et al., 2019; Eslahi et al., 2020; Wang et al., 2020), pharmaceutical (Li et al., 2018), environment (Xiang et al., 2018; Shoukat et al., 2019), energy (Li et al., 2014; Bu et al., 2018), sensing (Jasim et al., 2017; Farooq et al., 2020), electronics (Khan et al., 2015a; Dhar et al., 2019; Ul-Islam et al., 2020a), and several other fields (Aljohani et al., 2018; McCarthy et al., 2019a,b). For example, the presence of plentiful hydroxyl groups on its surface imparts hydrophilic nature to BC. Both the water holding and retention potential of BC are important from a medical perspective, especially in drug delivery (Li et al., 2018) and wound dressing (Di et al., 2017; Ciecholewska-Jusìko et al., 2020; Sajjad et al., 2020), where such properties are associated with the absorption of wound exudates and holding the drug for an extended time, thus greatly contributing to enhancing the therapeutic potential of BC. The free hydroxyl groups on the cellulose surface give rise to a binding module where the adsorption of the drug is driven through the formation of hydrogen bonding and van der Waals forces or through the electrostatic/aromatic interactions (Lombardo and Thielemans, 2019). Despite the broad-spectrum applications of BC in different fields, the lack of adhesion sites and low mechanical strength limit its direct use for different applications, thus necessitating the development of BC-based composites with other materials to impart such features to it.

*Aloe vera* represents one of the oldest plants known for its wound healing ability. The *Aloe vera* extract has been used in herbal and alternative medicine because of its antimicrobial, anti-inflammatory, and wound healing properties (Menda et al., 2014). Its gel mainly consists of water (99%) and mono- and polysaccharides (25% of dry weight). It contains 75 essential nutrients that play an important role in wound healing. The biocompatible feature of *Aloe vera* supports early epithelialization and develops a protective layer around the wound, thus leading to quick wound closure. Moreover, the moisturizing properties of *Aloe vera* gel accelerate cell proliferation, clot formation, and blood flow in the skin (Menda et al., 2014). The wound healing features of *Aloe vera* provide the base for the development of its composites with other biomaterials, including cellulose, chitosan, and alginate with optimized properties for biomedical applications (Pereira et al., 2013; Silva et al., 2013). Studies have reported that the addition of *Aloe vera* gel to the cellulose matrix increases its mechanical and biocompatible properties (Saibuatong and Phisalaphong, 2010; Godinho et al., 2016).

It has already been established that both the biocompatibility and mechanical properties of BC can be enhanced by developing its composites with appropriate materials (Khan et al., 2018). In the form of a hydrogel, BC contains only 1% cellulose, while approximately 99% is wet weight is comprised of water content; thus, any variation in water content leads to significant changes in its viscoelastic and electrochemical properties (Rebelo et al., 2018). The potential of different polymers like chitosan, gelatin, poly (vinyl alcohol), poly (3-hydroxybutyrate) as well as nanomaterials such as carbon nanotubes, zinc oxide, silver, TiO_2_, and Au, and montmorillonite clays have already been evaluated for enhancing the mechanical and biological features of BC (Ul-Islam et al., 2015; Ullah et al., 2016). Similarly, the addition of water-soluble materials could prove to be imperative in wound healing properties of BC, where moisture content is an essential factor. In an early study, BC-based composites with different contents of *Aloe vera* gel (0 to 50%) were developed through the *in situ* synthesis strategy by adding the *Aloe vera* gel to the BC-producing culture medium. The results showed that the addition of up to 30% (v/v) *Aloe vera* gel contributed to improving the structural morphology and associated properties of BC/*Aloe vera* (BCA) composite, however, further addition of *Aloe vera* to the culture medium not only decreased the BC pellicle formation but also affected the morphology, crystallinity, mechanical features, and water holding capacity and vapor transmission (Saibuatong and Phisalaphong, 2010). In contrast, the *ex situ* added *Aloe vera* would only be accommodated among the empty spaces in the fibril network without disturbing the structural morphology, and in turn, would contribute to enhancing the mechanical strength and other properties of BCA composite.

In the present study, we *ex situ* synthesized BCA composite by immersing the BC sheets in *Aloe vera* gel. The tensile properties of the developed BC-*Aloe vera* composite were determined, for the first time, through the full-field deformation and strain investigation by using the 3D-DIC technique. We provided insights into the changes in the structure of BC concerning the critical strain during the synthesis of the composite. Such information could further lead to the optimization of the composite structure for tailored mechanical features. Moreover, the developed BCA composite was evaluated for its potential applications in the biomedical and environment fields through cytocompatibility and heavy metal absorption analyzes, respectively.

## Experimentation

### Microbial Cell Culture

The BC-producing *Gluconacetobacter hansenii* PJK was cultured in a synthetic medium containing 10 g/L glucose, 7 g/L peptone, 10 g/L yeast extract, 0.2 g/L succinic acid, and 1.5 mL/L acetic acid. Additionally, 20 g/L agar was added to the synthetic medium for developing its solid cultures (Ul-Islam et al., 2014). The pH of the medium was adjusted to 5 by adding 0.1 N NaOH and autoclaved for 15 min at 121°C and 15 psi. The culture broth was prepared by inoculating few colonies of *G. hansenii* PJK grown on a solid culture plate into 100 mL of liquid broth in a 250 mL Erlenmeyer flask and incubated at 30°C under shaking at 200 rpm for 24 h.

### Production of BC Sheets

Bacterial cellulose sheets were produced in sterilized rectangular containers with dimensions 20 cm × 10 cm × 10 cm under static condition. Briefly, the culture medium was inoculated with 5–10% (v/v) freshly prepared *G. hansenii* PJK pre-culture and incubated at 30°C for 7–10 days under static condition, which produced about 7 mm thick BC sheets. The BC sheets were harvested from the air-medium interface, thoroughly washed, and boiled in 0.3 N NaOH to kill any live bacterial cells embedded in the BC matrix. The cell debris and residual medium contents were removed *via* frequent washing until the pH of the medium became neutral. The BC sheets were then stored in distilled water at 4°C for further use.

### Development of BCA

*Aloe collenetteae Lavranos* (*Aloe vera*) plant was collected from Salalah, Oman. The mature fresh leaves were picked and washed with distilled water. The thick epidermis of leaves was removed to collect the solid mucilaginous pulp, which was then grounded into a gel form and stored in a sterile container. Thereafter, BC and *Aloe vera* (BCA) hydrogel composite was *ex situ* developed at room temperature (i.e., 25°C) by dipping the 20 cm × 4 cm BC sheets in 300 mL *Aloe vera* gel for 2–3 days under shaking at 150 rpm in a beaker. The gel was adsorbed on the surface and impregnated into the BC matrix due to its porous geometry. The *Aloe vera* extract-loaded BC (BC-*Aloe vera*) membranes were refrigerated for further use. Both BC and BCA samples were air-dried and freeze-dried for desired characterization and use.

### FE-SEM Analysis

The morphological features of the freeze-dried pristine BC and BCA composite were determined through FE-SEM analysis using a Hitachi S-4800 & EDX-350 (Horiba) FE-SEM (Tokyo, Japan). Briefly, the samples were fixed onto a brass holder and coated with osmium tetroxide (OsO_4_) by a VD HPC-ISW osmium coater (Tokyo, Japan) for FE-SEM observation. FE-SEM analysis was conducted to observe the surface morphology and cross-sectional views of both pristine BC and BCA composite.

### Mechanical Testing

#### Material Preparation

**FIGURE 1:**
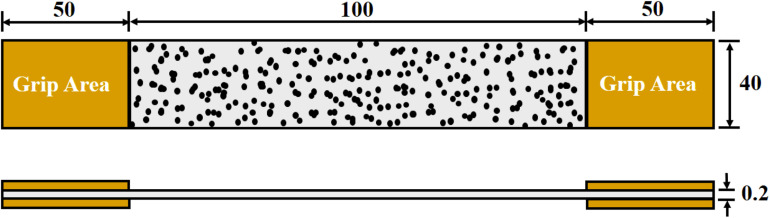
Illustration of dimensions of BC or BCA sample for tensile testing measurement *via* the 3D-DIC system. All dimensions are in mm. A set of three samples of each BC and BCA composite were prepared for tensile testing measurement.

The samples were prepared by cutting the sections from the smooth part of air-dried BC and BCA composite sheets with dimensions of 200 mm × 40 mm × 0.2 mm (Figure 1). The white and black paints were used to create the speckled pattern on the surface of the samples to measure the full-field displacement and strain using the 3D-DIC system.

#### Tensile Test Setup

**FIGURE 2:**
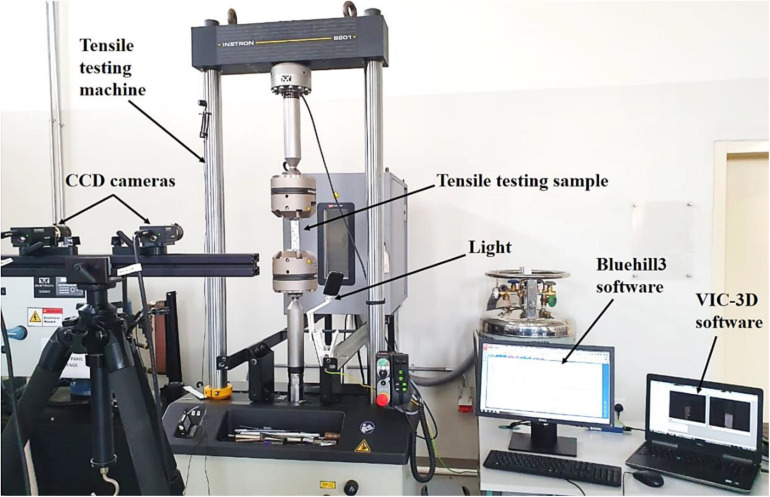
The tensile test setup with a 3D-DIC system for the determination of mechanical properties of objects.

The tensile test was carried following a previously reported protocol (Abbassi and Ahmad, 2020; Jebri et al., 2020). Briefly, to compute the full-field strain, the 3D-DIC system was used, consisting of two high-resolution CCD cameras for image acquisition and a VIC-3D software for computing the DIC results. To correlate the two images, the first image of the object is taken as the reference, and it is correlated with the next image. The DIC system selects two subsets of pixels from the reference and deformed images having central point *P* and *P*^∗^, respectively. The system then calculates the displacement by taking the difference in the position of these two central points. An incandescent lamp is used to avoid the noise in the images. An Instron testing machine was used (Bluehill 3 software, the capacity of 100 kN) to measure the tensile properties of pristine BC and BCA composite. A constant loading rate of 1 mm/min was selected to perform all tests, and the tensile tests for pristine BC and BCA composite were performed in triplicate. A complete experimental setup used to perform the analysis of the tensile properties using the tensile testing machine and 3D-DIC system for the full-field strain shown in Figure 2.

### Water Holding and Retention Analyses

The BC and BCA composites were tested for their water holding capacity (WHC) and water retention time (WRT). Briefly, the samples were cut into 5 cm × 3 cm rectangular pieces and freeze-dried at −80°C and 0.5 torr pressure for 48 h using the Uni-Freeze FD 8 model (DAIHAN Scientific). The freeze-dried sheets in a set of three samples were immersed in distilled water under static conditions at room temperature. By reaching the complete wet state (stabilized wet weight), the samples were analyzed for WRT by measuring their weights at various time intervals at ambient temperature until complete drying. The WHC was calculated by using the below equation:

$$W\,H\,C=\frac{\mathrm{Mass}\,\mathrm{of}\,\mathrm{water}\,\mathrm{removed}\,\mathrm{during}\,\mathrm{drying}\,\left(g\right)}{\mathrm{Dry}\,\mathrm{weight}\,\mathrm{of}\,\mathrm{BC}\,\mathrm{sample}\,\left(g\right)}$$

### Absorption and Desorption Studies

For heavy metal absorption analysis by the pristine BC and BCA composite hydrogels, different metal salts including Zn(NO_3_)_2_, Cu(NO_3_)_2_, Co(NO_3_)_2_, and Fe(NO_3_)_3_ were dissolved in deionized water at a concentration of 10 mg/L (10 ppm). Thereafter, 1 g each of freeze-dried BC and BCA composite was cut into small pieces and loaded into the flasks containing 250 mL of heavy metal solution. The samples were incubated overnight under stirring at 200 rpm at 25°C. For analysis, a 10 mL solution from each flask was taken and tested for residual metal ion concentrations using the atomic absorption spectrophotometer. For desorption studies, the harvested samples were placed in flasks containing 250 mL deionized water and stirred overnight at 200 rpm at 25°C. Finally, a 10 mL solution was taken from the flasks and analyzed for desorbed metal ion concentrations using the atomic absorption spectrophotometer. The adsorption studies for both pristine BC and BCA composite were carried out in triplicate.

### Cell Attachment and Proliferation Analysis

The cytocompatibility of pristine BC and BCA hydrogels was evaluated against osteoblast cells (MC3T3-E1 ATCC). Briefly, 10 mm diameter samples were sterilized by autoclaving at 121°C and 15 psi for 15 min and immersed in DMEM (Dulbecco’s modified Eagle’s medium) containing 10% FBS and 1% penicillin-G-streptomycin for 24 h. After pre-treatment, the samples were carefully placed in six-well plates. Thereafter, the cells were seeded on all samples at a density of 3 × 10^5^ cells/well and incubated at 37°C and 5% CO_2_ for 1 and 2 days. After incubation, the supernatant was discarded, and the samples were washed a few times with PBS and then fixed with 3% glutaraldehyde for 20 min. The cell adhesion on the hydrogel scaffolds was examined through FE-SEM analysis. The proliferation of MC3T3-E1 cells was determined in a 24-well plate through the ELISA method according to the manufacturer’s instructions (Roche Molecular Biochemicals) (Haider et al., 2014; Ul-Islam et al., 2014). This method is based on the measurements of 5-Bromo-2-deoxyuridine (BrdU), which is up taken by the dividing cells during the DNA synthesis.

### Statistical Analysis

All results are expressed as means ± SD for each experimental condition. Statistical analyses were performed using the two-tailed student’s *t-test*. The difference between pristine BC and BCA composite was considered statistically significant at ^∗^*p <* 0.05.

## Results and Discussion

### Composite Synthesis and Characterization

**FIGURE 3:**
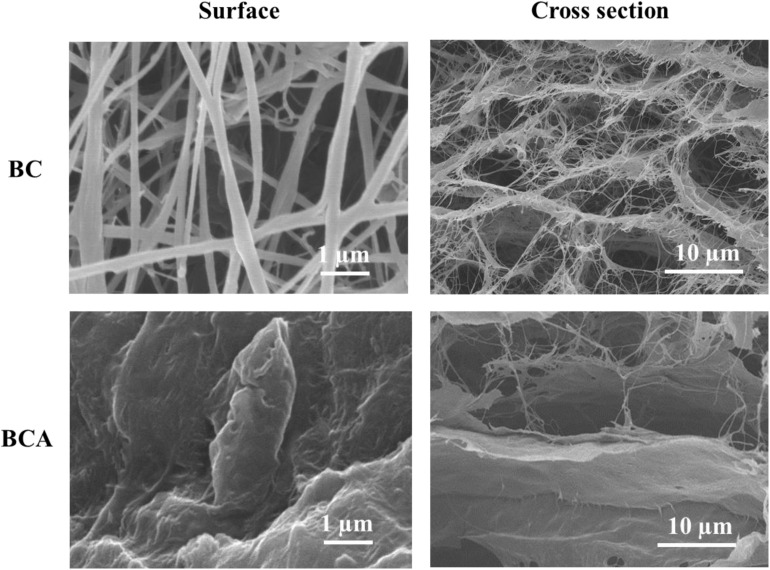
Field-emission scanning electron micrographs of the surface and cross-sectional views of pristine BC and *ex situ* developed *Aloe vera*-loaded-BC composite (i.e., BCA) containing 40 wt.% *Aloe vera* gel.

The BC-based composites are commonly prepared through *in situ*, *ex situ*, and solution casting methods. Earlier reports of developing composites of BC with *Aloe vera* used the *in situ* strategy where *Aloe vera* gel was added to the culture medium of BC-producing microorganism. The added *Aloe vera* gel was entrapped among the growing cellulose fibrils (Saibuatong and Phisalaphong, 2010; Godinho et al., 2016), however, the *in situ* addition of *Aloe vera* above 30% (v/v) of the total volume of culture medium increased the viscosity of the medium, which consequently resulted in reduced BC production and altered morphology and mechanical features. The presence of high content of *Aloe vera* gel increased the viscosity of the medium and interrupted the oxygen supply to the growing microbial cells, thus leading to their retarded growth and ultimately resulted in low BC production with compromised features (Saibuatong and Phisalaphong, 2010). Keeping the high viscosity-induced retarded growth of bacterial cells during the *in situ* composite development strategy, we adopted the *ex situ* approach for the development of BC-*Aloe vera* composite hydrogel in the present study, where *Aloe vera* gel was impregnated into the matrix of the already produced BC. The degree of *Aloe vera* attachment to the surface and impregnation into the BC matrix and successful development of BCA composite was confirmed through FE-SEM microscopic observation of freeze-dried BC and BCA (Figure 3). The *ex situ* added *Aloe vera* gel was adsorbed not only on to the surface but also impregnated into the BC matrix. It was observed that the volume of *Aloe vera* gel did not change after 72 h immersion of BC pieces, indicating the saturation of accessible empty spaces in the fibrous network of BC. The *ex situ* addition of *Aloe vera* gel did not affect the fibrous morphology of BC as evident from the FE-SEM micrographs (Figure 3). Dry-weight analysis showed that the *Aloe vera* gel occupied 40% weight in the BCA composite.

The surface and cross-sectional views of the FE-SEM micrograph of pristine BC show a fibrous and porous network, indicating an ideal scenario for the impregnation of *Aloe vera* gel solution into its matrix. FE-SEM micrographs of both the surface and cross-sectional views of BCA composite shows the successful impregnation of *Aloe vera* into the BC matrix, where it got attached to the cellulose fibrils and covered the almost entire surface. The cross-sectional images also indicate the impregnation of *Aloe vera* gel among the layers of cellulose fibers and occupied the empty spaces in the matrix. This strong attachment could be attributed to the intermolecular hydrogen bonding between the hydroxyl groups on BC and amine and hydroxyl groups of *Aloe vera* (Saibuatong and Phisalaphong, 2010). Such strong interactions could contribute to enhancing the physico-mechanical properties of BCA composites.

### Mechanical Strength of BCA Composite Hydrogel

For mechanical testing analysis, air-dried samples were used where the weak reversible hydrogen bonding among the cellulose fibrils is converted to strong irreversible hydrogen bonding upon the elimination of water content and leads to compact packing of the fibrils (Ul-Islam et al., 2013). The compact fibrils and strong hydrogen bonding restrict the mobility of polymer chain and enhance the toughness of BC and BC-based composites (Velmurugan and Mohan, 2009), which could offer more resistance to the applied external force and resist any structural changes in their structure. Therefore, it is expected that air-dried BC and BCA could offer high mechanical strength as compared to the freeze-dried samples.

**FIGURE 4:**
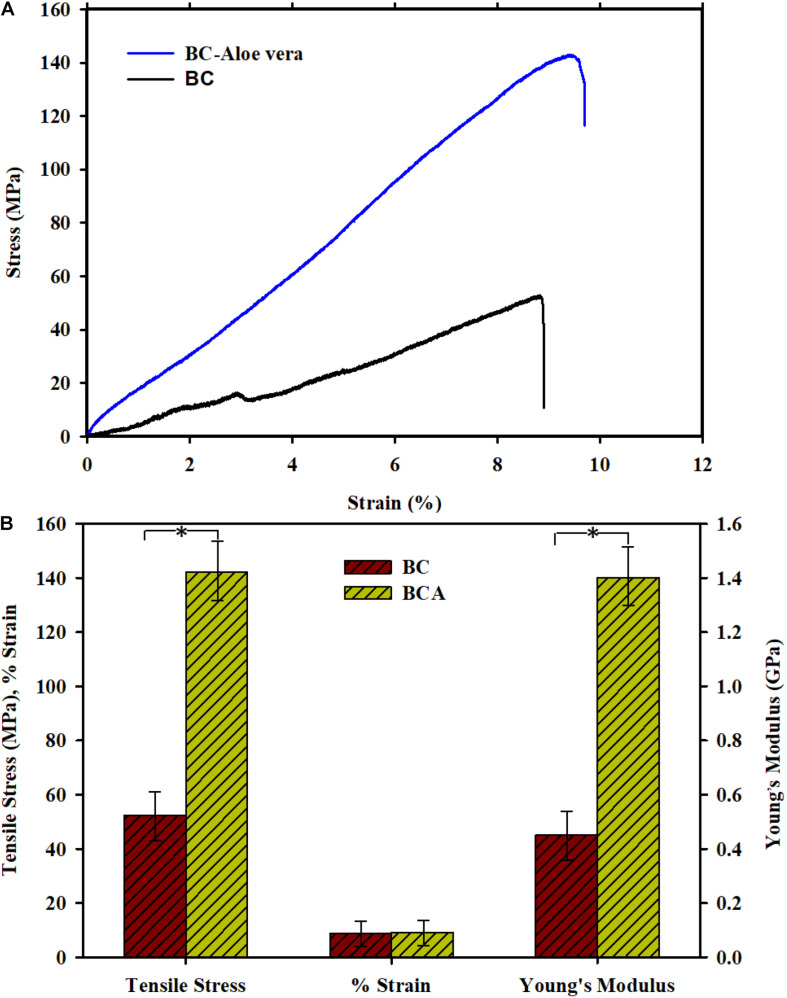
Mechanical properties of pristine BC and *ex situ* developed *Aloe vera*-loaded-BC composite (i.e., BCA). **(A)** Stress-strain curve and **(B)** Tensile strength, % strain, and Young’s modulus of BC and BCA composite. Asterisk (^∗^) indicates a *P*-value of <0.05.

The mechanical testing results shown in Figure 4 indicate that BCA composite possessed better mechanical strength than pristine BC. The pristine BC and BCA composite showed a tensile strength of 54.3 and 142.4 MPa and Young’s modulus of 0.45 and 1.4 GPa, respectively. These results show a 3-fold increase in the mechanical properties of BCA composite as compared to the pristine BC. Moreover, the elasticity of BCA and BC hydrogels was found to be almost of the same strength. The increase in the mechanical features of BC, when composited with other materials, has been reported in several studies (Kim et al., 2010; Wang et al., 2018). In a study, the addition of 0 to 30% (v/v) *Aloe vera* gel resulted in a 1.6-fold increase in the tensile strength and elongation break of *in situ* prepared BCA composite, however, further addition of *Aloe vera* gel into the BC matrix resulted in reduced mechanical properties due to the disruption of BC fibril structure and arrangement during the *in situ* synthesis process (Saibuatong and Phisalaphong, 2010). The use of the *ex situ* composite synthesis strategy in the present study contributed to maintaining the undisturbed structural features of BC, which aided in enhancing its existing features and strengthening of fibrils upon the incorporation of *Aloe vera* gel. The maintenance of the original fibrous structure of BC upon the addition of *Aloe vera* gel could be the possible reason that even a high concentration (i.e., 40 wt.%) of *Aloe vera* gel in the BC matrix contributed to a 3-fold increase in the tensile strength.

**FIGURE 5:**
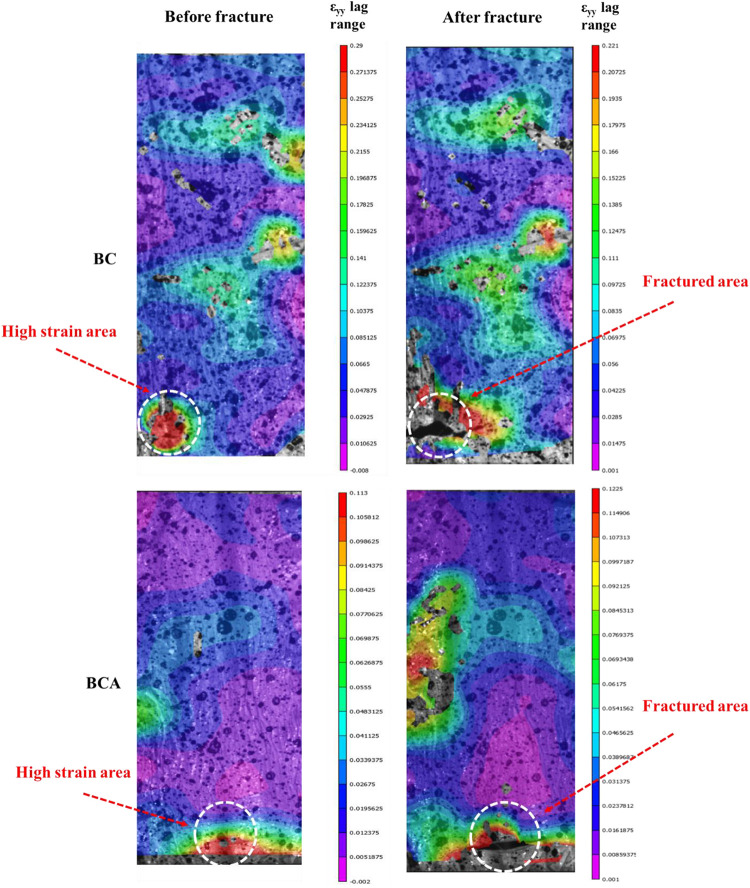
Mechanical behavior of pristine BC and *ex situ* developed *Aloe vera*-loaded-BC composite (i.e., BCA) determined through the 3D-DIC system. The deformation of samples was determined before and after a fracture. The arrows indicate the high strain and fractured areas in both pristine BC and BCA composite.

The mechanical strength of the BCA composite was further determined through a 3D-DIC system *via* full-field deformation to provide insight into the BC structure with respect to the critical strain during the synthesis of the composite. The 3D-DIC is a modern but simple operational optical technique with a high level of precision in the field of engineering research. It is mainly used to obtain the full-field displacement and strain without any physical contact with the deforming object under any kind of loading. Although many researchers have used the 3D-DIC system for failure mechanism analysis of different structures and materials (Abbassi and Ahmad, 2020; Jebri et al., 2020), its application in BC research has been limited. In this study, the 3D-DIC system was used to locate the critical strain, and the fracture process zones for both pristine BC and BCA composite samples under tensile loading, and the results are shown in Figure 5. The image for pristine BC shows that the bottom left corner of the sample is under high strain (indicated by an arrow on the left side), and the sample deformed from the same location is shown on the right side (indicated by an arrow). From the deformed image of pristine BC, it is clear that further tensile loading will continue the deformation in the upward and horizontal direction from micro to large scale cracks. The results from the 3D-DIC system for the BCA sample, before and after the fracture, are also shown in Figure 5, indicating that the sample is comparatively under less strain (indicated by arrows). The respective deformed image from the same location in the BCA composite is shown on the right side (indicated by an arrow). It is clear from the left side of Figure 5 that the samples initially failed from the bottom in the middle, indicating this point as the location of critical strain. The prediction of the 3D-DIC system was found accurate as the sample started to deform from the same location, indicating that it will continue to deform in the horizontal direction if a further tensile load is applied.

### Absorption of Water and Heavy Metals by BCA Composite

**FIGURE 6:**
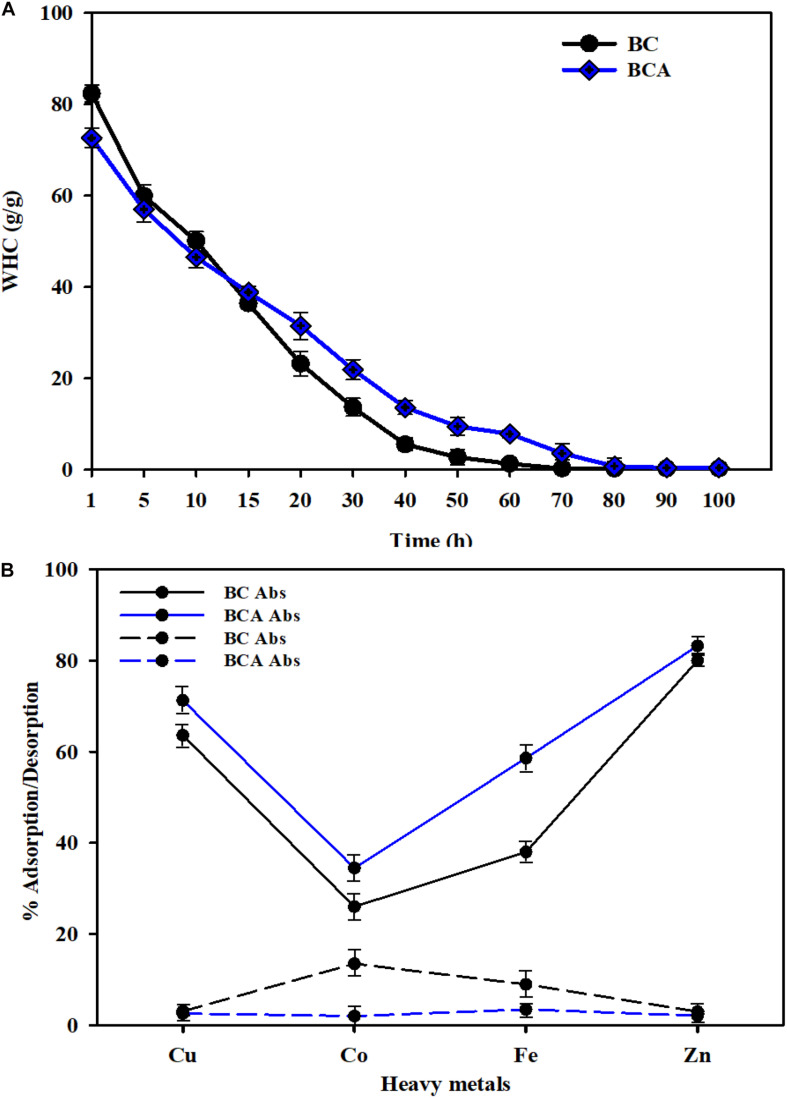
Absorption analysis of pristine BC and *ex situ* developed *Aloe vera*-loaded-BC composite (i.e., BCA). **(A)** Water holding capacity **(B)** heavy metal absorption analysis for all samples was carried out at room temperature. Error bars indicate that the results are the mean from three independent experiments.

The porous surface and hydrophilic nature of BC impart tremendous liquid and solid adsorption and water holding capabilities to BC. Several studies have reported that BC can hold water from a few to more than a hundred times its dry weight (Ul-Islam et al., 2013). The WHC of BC varies with its structural and processing features depending upon its surface area, pore size, and pore volume. The results for WHC of pristine BC and BCA composite are shown in Figure 6A. The results show that compared to the pristine BC holding 84 times more water than its dry weight, the BCA composite only held 73 times more water than its dry weight, indicating that the impregnation of *Aloe vera* gel to BC network reduced its WHC. The reduced WHC of BCA could be attributed to the reduction in the porosity of BC sheets and the unavailability of free hydroxyl groups in BC due to their interaction with those in the *Aloe vera* gel. Interestingly, the BCA composite retained water for a much longer time compared to the pristine BC, which could be attributed to the hydrophilic nature of *Aloe vera* gel. Figure 6A illustrates that pristine BC lost almost 90% of the water after 40 h, whereas BCA lost the same amount of water in 70 h. The slow WRT from BCA composite is an important feature for its application in drug delivery, chronic wound healing, and prolonged skin moisturizing to maintain a moist environment for cosmetic application (Khan et al., 2015b; Di et al., 2017).

The porous structure of BC plays an important role in absorbing heavy metals from aqueous solutions (Jin et al., 2017). To date, BC and other forms of cellulose have been extensively used in developing filters, where the primary aim is the absorption of unwanted and toxic materials from wastewater (Ul-Islam et al., 2016). Owing to its 3D nano-reticular microenvironment, BC could prove to be a better option for the absorption of heavy metals as compared to the crystalline cellulose (Chen et al., 2009). In the present study, the heavy metals absorption capabilities of both BC and BCA were evaluated against heavy metals, including Cu, Co, Fe, and Zn, and the results are shown in Figure 6B. The results show that both pristine BC and BCA composite effectively absorbed heavy metals from water, nevertheless, to different levels. The absorption ability of the BCA composite was better than pristine BC. Furthermore, the strength of the absorbed particles with cellulose fibrils was determined through desorption analysis, where an almost negligible desorption rate was observed with pristine BC and BCA composite. The BC-based composites with metal absorbing capabilities have already been reported where BC composite with poly (ethylene glycol) (BC-PEG) showed high potentials of Cu^2+^ and Pb^2+^ ions adsorption from aqueous solution (Chen et al., 2009). In another study (Chen et al., 2020) developed the composite of BC with attapulgite that effectively absorbed Pb^2+^, Cu^2+^, and Cr^6+^, along with anionic organic dyes. Considering the structural features of BC, it can be concluded that this biopolymer could be utilized as a green material for developing composites for absorbing pollutants and toxic materials from wastewater, air, and other samples.

### Cell Adhesion and Proliferation Analysis

**FIGURE 7:**
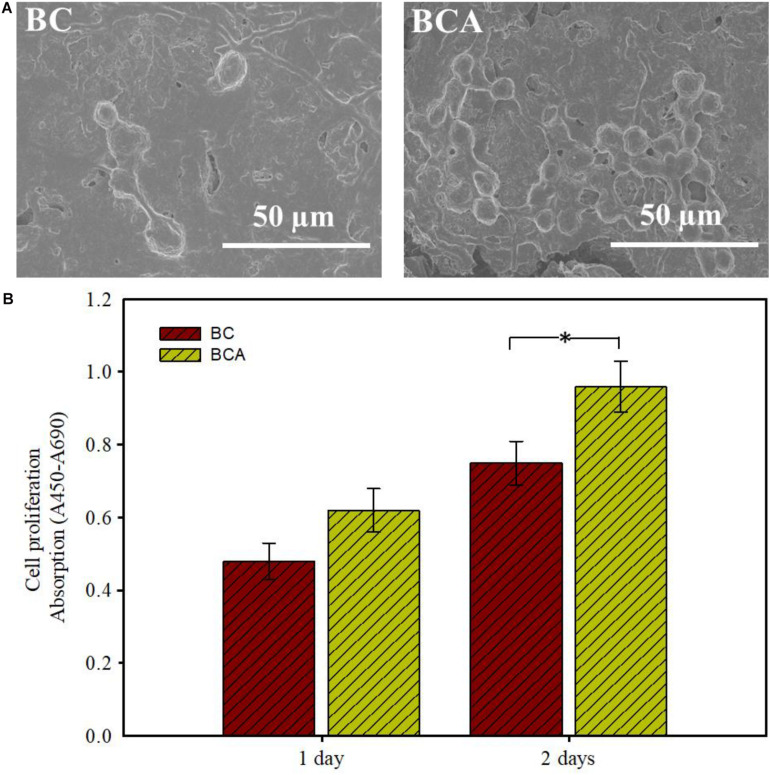
Cytocompatibility analysis of pristine BC and *ex situ* developed *Aloe vera*-loaded-BC composite (i.e., BCA). **(A)** The adhesion and structural morphology of MC3T3-E1 cells on both samples was observed under FE-SEM. **(B)** The proliferation of MC3T3-E1 cells on the samples was determined by measuring the absorption after 1 and 2 days. The asterisk (^∗^) indicates a *P*-value of <0.05.

To validate the feasibility of pristine BC and BCA composites in wound healing and related biomedical applications, it is important to observe their cell adhesion capabilities against animal cells. In the present study, the cytocompatible and non-toxic nature of pristine BC and BCA composite hydrogel was evaluated by determining their ability to support the adhesion and proliferation of MC3T3-E1, and the results are shown in Figure 7. The cell adhesion observed *via* FE-SEM revealed successful adhesion of MC3T3-E1 cells on both pristine BC and BCA composite (Figure 7A). Further, the MC3T3-E1 cells attained round morphology after 12 h on both BC and BCA composites displaying minor signs of their shape extension and formation of filopodia. Compared to the pristine BC, the MC3T3-E1 cells proliferated more effectively on BCA composite, indicating the improved cytocompatibility of BC upon impregnation of *Aloe vera* gel. The examination of cell proliferation illustrated appreciably high levels of BrdU absorption when cultured on both BC and BCA samples (Figure 7B). A significant increase in cell proliferation was observed on BCA composite as compared to the pristine BC after 1 and 2 days. These observations are in accordance with an earlier study (Godinho et al., 2016). Godinho et al. (2016) reported that BC-*Aloe vera* composite possess offered high cytocompatibility than pristine BC, where the viable animal cells fully covered the composite surface, indicating a strong adhesion and proliferation. The high level of cell adhesion capabilities of BCA composites could be attributed to the nitrogen-containing compounds in *Aloe vera* gel. Overall, the results of the current study show the potential of BCA composite with high mechanical strength as a remarkable scaffold for wound-healing applications.

## Conclusion

A composite of BC with *Aloe vera* gel (BCA) was successfully developed through the *ex situ* composite synthesis strategy. The employed *ex situ* approach of impregnating *Aloe vera* gel into the BC matrix overcame the limitation associated with the *in situ* synthesis strategy and did not disturb the fibrous morphology, and contributed to enhancing the mechanical strength of BCA composite. The position of critical strain or stress concentration points were identified in the developed BCA composite by using the 3D-DIC system. The developed BCA composite demonstrated a high level of heavy metal absorption and supported the adhesion of animal cells, indicating its multipurpose applications in environmental and biomedical sectors, respectively. It can be concluded that the synthesized BCA composite with higher mechanical, cytocompatibility, and absorption properties could be used as a multipurpose material for applications in biomedical, environment, and other fields. Further, *in vivo* studies to evaluate the detailed biocompatibility for wound healing and tissue regeneration ability as well as drug loading capacity and release profile are warranted for practical tissue engineering applications of the developed BCA composite. Similarly, the utilization of BCA-composites as catalysts, adsorbents, and membrane filters and applying them to wastewaters, air, soil, and other samples wound further demonstrate their potential environmental applications.

## Data Availability Statement

The original contributions presented in the study are included in the article/supplementary material, further inquiries can be directed to the corresponding author.

## Author Contributions

FA, AF, and SY: methodology, investigation, and characterization. NS, MdA, SM: writing – original draft, writing – review, and editing. MU-I and MU: supervision, investigation, methodology, writing – review, and editing. All authors contributed to the article and approved the submitted version.

## Conflict of Interest

The authors declare that the research was conducted in the absence of any commercial or financial relationships that could be construed as a potential conflict of interest.
